# Prognosis of transarterial chemoembolization-sorafenib compared to transarterial chemoembolization-alone in hepatocellular carcinoma stage C: a systematic review

**DOI:** 10.1186/s43046-024-00224-4

**Published:** 2024-05-27

**Authors:** Rahmad Mulyadi, Irsan Hasan, Prijo Sidipratomo, Pungky Permata Putri

**Affiliations:** 1 Department of Radiology, Faculty of Medicine, Universitas Indonesia, Jakarta, Indonesia; 2 Department of Internal Medicine, Faculty of Medicine, Universitas Indonesia, Jakarta, Indonesia

**Keywords:** Hepatocellular carcinoma, Transarterial chemoembolization, Sorafenib, Neoplasm staging, Survival analysis

## Abstract

**Background:**

This systematic review aims to compare the prognosis of treatment transarterial chemoembolization (TACE) combined with sorafenib and TACE-alone in patients with hepatocellular carcinoma (HCC) with Barcelona clinic liver cancer-stage C (BCLC-C).

**Materials and methods:**

A systematic search was conducted on five electronic databases: PubMed, ScienceDirect, Cochrane, Embase, and Scopus. Studies were included if they compared overall survival (OS) of TACE-Sorafenib to TACE-alone in patients with HCC BCLC-C within the 2019–2023 timeframe. We excluded studies consisting of conference abstracts, letters, editorials, guidelines, case reports, animal studies, trial registries, and unpublished work. The selected articles were evaluated from August 2023 to September 2023. The journal’s quality was assessed with NOS for a non-randomized controlled trial.

**Results:**

This systematic review included four studies following the Preferred Reporting Items for Systematic Review and Meta-analysis (PRISMA). All four studies compared the OS of 401 patients with TACE-sorafenib to TACE-alone. Two studies compared time-to-progression (TTP), one study compared progression-free survival (PFS), and two studies compared disease control rate (DCR). There were various population criteria, TACE techniques used, risk factors, follow-up time, and adverse events. The collected evidence generally suggested that the combination of TACE-sorafenib is superior compared to TACE-alone. Due to a lack of essential data for the included study, a meta-analysis couldn't be performed.

**Conclusion:**

The results of this systematic review suggested that TACE-sorafenib combination therapy in patients with HCC BCLC-C improves OS superior compared to TACE-alone, without a notable increase in adverse events.

**Supplementary Information:**

The online version contains supplementary material available at 10.1186/s43046-024-00224-4.

## Background

Hepatocellular carcinoma (HCC) is a primary liver cancer in 75 to 85% of all cases. Often, HCC is detected when it is already an advanced stage, and limited treatment options are available for such cases [1].

HCC staging plays a key role in determining treatment strategies and predicting overall prognosis. Among various prognostic systems, the Barcelona Clinic Liver Cancer (BCLC) system shows the strongest correlation with patient outcomes [2].

BCLC classified patients into five stages of HCC, classified as 0, A, B, C, or D. Provided treatment recommendations based on three critical prognostic factors which are tumor characteristics, liver function, and performance status. BCLC-C, classified patients by symptoms, macrovascular invasion, or extrahepatic dissemination. Palliative interventions are recommended, typically advised transarterial chemoembolization (TACE) or sorafenib for BCLC B and C stages. A study conducted by Rashed et al. stated that late-stage HCC diagnoses in Egypt are associated with median overall survival (OS) between 6 and 20 months [2]. In advanced-stage cases where palliative treatments are applied, the median post-diagnosis survival spans 6 to 12 months [1, 3].

The systematic review focused on narrowing the study population only to BCLC Stage C patients as the study population is critical for several reasons. Narrowing the study population allows for a more precise evaluation of treatment outcomes and reduces confounding factors. This approach is clinically relevant as it addresses the unique challenges associated with the management of advanced HCC and provides evidence-based guidance for treatment decisions in these critical cases. Hopefully, this approach helps the management of healthcare resources, given that stage C BCLC patients have limited treatment options. This systematic review wants to offer valuable insights that can be useful for clinical practice and further research, ultimately improving the care and outcomes of individuals with advanced HCC.

Although the BCLC system is effective in predicting survival in Western and Asian populations, it is not the preferred classification system in Asia. Treating advanced HCC varies across Asian countries. Therapies such as external radiotherapy, intra-arterial and systemic chemotherapy, and TACE are often used despite limited evidence for their effectiveness [4].

In Europe and the USA, guidelines used the BCLC staging system. The recommendation is to treat these patients with molecular-targeted drugs like sorafenib and lenvatinib. Meanwhile, experts in Southeast Asian countries, advocate for a multidisciplinary approach that includes surgery, TACE, radiation therapy, and molecular-targeted drugs, for a better outcome for HCC patients [5].

sorafenib and TACE are both recommended treatments for advanced HCC that have been used in certain regions. The effectiveness of this combined approach remains to be determined. An intriguing question arises about its safety and efficacy when compared to TACE alone.

## Methods

### Search strategy

This review follows PRISMA guidelines [6]. The investigation was started on 2nd August 2023. This systematic review was executed across five electronic databases: PubMed, ScienceDirect, Cochrane, Embase, and Scopus. The objective was to identify all relevant studies on overall survival (OS) prognosis after TACE therapy with or without combination sorafenib therapy for HCC BCLC stage C. The search keywords used were (“Hepatocellular carcinoma”) OR (“HCC”) AND (“Stage C”) AND (“Transarterial chemoembolization”) AND (“Sorafenib”) AND (“Prognosis”). The investigation and evaluation of the chosen articles was carried out between 2nd August and 30th September 2023.

### Eligibility criteria

The inclusion criteria for the included study in this review were (1) the population comprised patients with HCC with BCLC, Stage C. (2) The index test involved TACE with a combination of sorafenib therapy. (3) The outcome included the survival prognostic TACE with and without combination sorafenib therapy. (4) The journal range is within 5 years (2019–2023). The search strategy excluded conference abstracts, letters, editorials, guidelines and consensus statements, systematic reviews or meta-analyses, case reports, literature reviews, xenograft/animal model studies, trial registries, and unpublished studies. There were no language restrictions in the search strategy.

### Study selection

Four different reviewers investigated the literature and study selection. The selection process was reviewing titles and abstracts in five databases: PubMed, ScienceDirect, Cochrane, Embase, and Scopus, removing duplicate or similar articles using EndNote 20 software, and evaluating full articles. Additionally, we examined the references cited in the selected study to identify any additional relevant research. Any disagreements were resolved through discussions that included all the authors.

### Data extraction and study-quality assessment

The information collected included the following. (1) The study characteristics included author information, study year, the country where the study was conducted, number of patients, and measured median OS (2) The patient characteristics included age, HCC BCLC-C, no history of other malignancy, TACE with or without combination with sorafenib as therapy. (3) Regarding response data, responders were defined as patients with overall survival of the therapy. We intended to compute pooled hazard ratios if data were accessible from the included study. However, out of the four included studies using Kaplan-Meier (KM) and log-rank tests, only Ren et al. provided HR and CI. The other studies lacked the number at risk and supplementary data, thus we couldn't perform a meta-analysis.

### Risk of bias and applicability

The quality and risk of bias of the included study in this systematic review were evaluated using the Newcastle-Ottawa Scale for non-randomized study [7]. Stars were assigned to each domain. The grading thresholds for converting the NOS to Agency for Healthcare Research and Quality (AHRQ) standards were determined as follows [8]

#### Good quality

The stars in the selection domain reached 3 or 4 stars, the comparability domain received 1 or 2 stars, and there are 2 or 3 stars in the outcome domain.

#### Fair quality

The stars in the selection domain reached 2, and the comparability domain got 1 or 2 stars, and there are 2 or 3 stars in the outcome domain.

#### Poor quality

If there were 0 or at least 1 star in the selection domain, no stars in the comparability domain, and no or 1 star in the outcome domain.^8^

## Results

### Study selection

**Fig. 1:**
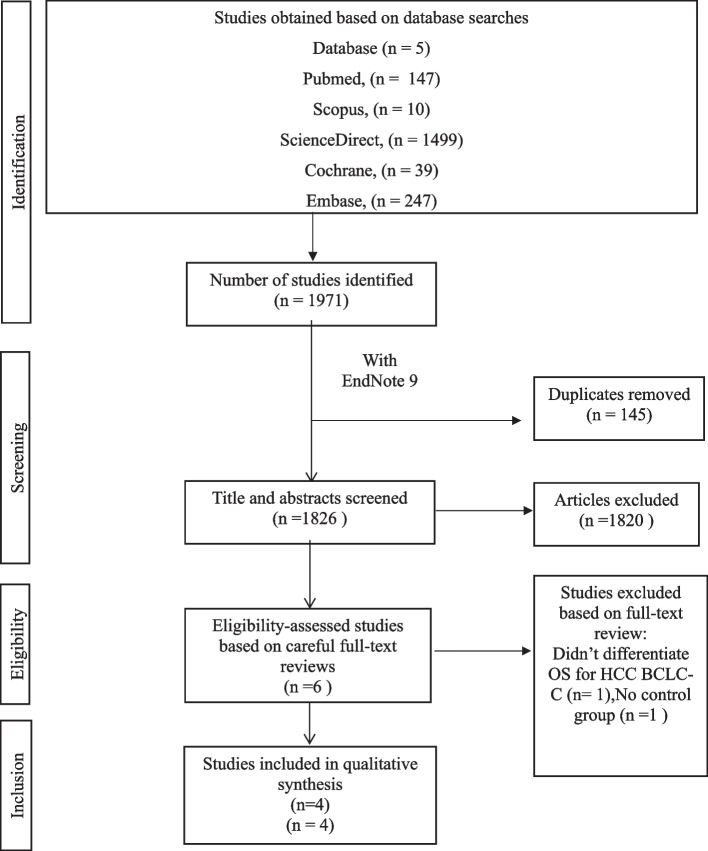
Flow chart. Description of reasons for including/excluding research

In this systematic review, we ultimately included four studies from an initial pool of 1971 identified across five databases (PubMed, ScienceDirect, Cochrane, Embase, and Scopus). After removing 145 duplicate entries, we subjected 1826 studies to further evaluation. Among these, 1820 studies were excluded after reviewing their titles, abstracts, and free access to journals. One study was excluded because did not differentiate OS for specifying in BCLC-C. One study was excluded because did not have a control group. Subsequently, we conducted a detailed assessment of the full text of 4 studies. Any study that did compare the overall survival of TACE-sorafenib and TACE-alone and studies that did not mention BCLC-C were excluded. Ultimately, four studies were found to meet the eligibility criteria. The process of creating this systematic review is visually represented in Fig. 1. PRISMA Flow Chart. Surprisingly, only one study presented the HR and CI of the Kaplan-Meier *p* values. None of the Kaplan-Meier data provided the number at risk or provided supplementary data so this systematic review could not perform a meta-analysis.

### Quality assessment of the included study

The quality of the study was evaluated following the Newcastle-Ottawa Scale checklist. Table 1 shows the risk of bias and applicability of the selected study. The quality of the included study was deemed to be good quality. In all investigations, the reasons for patient exclusion were explicit and appropriate. In general, however, the selected individuals were heterogeneous regarding the primary malignancy and the given sorafenib time. The treatment-response evaluation period was generally less than 3 months, indicating an early treatment-response evaluation.

**Table 1:** Newcastle-Ottawa Quality Assessment

Study	Selection				Comparability	Outcome			Quality
	Representativeness of exposed cohort	Selection of nonexposed	Ascertainment of exposure	Outcome not present at the start	The study controls for treatment	Assessment of outcome	Adequate follow-up length	Adequacy of follow-up	
Liu, S (2022)	c	*	*	*	**	*	*	*	Good
Liu, CK (2020)	c	*	*	*	**	*	*	*	Good
Ren (2019)	c	*	*	*	**	*	*	*	Good
Patidar (2020)	c	*	*	*	**	*	*	*	Good

*c* selected group; *, one star; **, two star; *** three star

Good quality: if the stars in the selection domain reached 3 or 4 stars, 1 or 2 stars in the comparability domain, and 2 or 3 stars in the outcome domain

Fair quality: if there were 2 stars in the selection domain, 1 or 2 stars in the comparability domain, and 2 or 3 stars in the outcome domain

Poor quality: if there were 0 or 1 star in the selection domain, 0 stars in the comparability domain, or 0 or 1 star in the outcome domain

### Studies characteristics

This systematic review included four studies comparing the OS of patients with treatment TACE-sorafenib and TACE-alone. These studies included 401 patients with HCC BCLC-C. The features of each study are presented in detail in Table 2. All four retrospective studies were published between 2020 and 2023: China and India. All patients that were included in the studies are patients over 18 years old with HCC BCLC-C [9–12].

**Table 2:** Compared study of overall TACE-sorafenib and TACE-alone

No.	Study (year)	Country	Study design	Total sample BCLC C	TACE-sorafenib	Control group (TACE-alone)	Sorafenib dose	OS TACE-sorafenib	OS TACE-alone	*P* value
1	Liu S (2022)	China	Retrospective study	120	60	60	On the 4th to 7th day after TACE, 400 mg twice a day	22.9 months	12.1 months	*P* = 0.016
2	Liu CK (2020)	China	Retrospective study	75	35	40	On the 4th day after TACE treatment. Sorafenib 400 mg	13.6 months	6.5 months	*P* = 0.041
3	Ren (2019)	China	Retrospective Study	128	31	97	On the 3th–5th day after TACE, 400 mg twice daily	Before matching 15.8 ± 2.0 months After matching 15.8 ± 2.0 months	Before matching 7.8 ± 1.1 months after matching 8.3 ± 1.4 months	Before matching *P* = 0.003 after matching *P* = 0.016
4	Patidar (2021)	India	Retrospective study	78	47	31	TACE and sorafenib 400 mg twice daily are given together	10.15 ± 2.8 months	7.81 ± 1.4 months	*P* < 0.001

*OS* overall survival, *TACE* transarterial chemoembolization

The inclusion criteria in these studies represent a concerted effort to establish a homogeneous patient population for research studies. Patients with advanced HCC, good liver function, and good performance status parameters are eligible for inclusion across all four studies. Most importantly, a definitive diagnosis of HCC is an essential requirement. Patients must have their HCC confirmed through various ways, such as histological examination, cytological tests, or imaging methods that adhere to established diagnostic criteria.

Patients included in the study must be at an advanced stage of the disease. They should be classified under the BCLC-C. This staging system helps ensure that the patients considered for the studies have similar disease severity. All studies mention the necessity for patients to have acceptable liver function. The Child-Pugh classification, a well-established tool in hepatology, is commonly used to assess this. The Eastern Cooperative Oncology Group (ECOG) performance status in all studies helps gauge patient’s overall health and functional capacity. The inclusion is consistent with ECOG scores of 0 or 1.

Ren et al. included all unresectable HCC cases in their study. The researchers used the PSM cohort method to match samples and minimize bias. The OS was measured HCC BCLC-C and B (matched sample and unmatched) [9]. None of the studies included patients who have undergone liver transplantation. This is a deliberate choice, as liver transplantation represents an entirely different treatment approach for patients with HCC. The exclusion of transplant recipients ensures that the study populations remain focused on the specific interventions under investigation, such as TACE and combination therapies, rather than the outcomes of transplantation.

All studies uniformly exclude patients with concurrent malignancies [9–12]. One study excluded patients with a history of resection [12]. This exclusion criterion ensures that the study’s populations comprise solely individuals with primary HCC. Excluding patients with other primary malignancies helps maintain the integrity of the research by eliminating potential confounding factors that may arise from other malignancies.

Liu, S. et al. Unlike the other studies, excluding patients with bone marrow and heart dysfunction, without specifying the cardiac issues [11]. Patidar et al. explicitly excluded patients with severe coagulopathy and demonstrated more effort to avoid bleeding risks associated with the procedure [12].

### Technique of TACE and sorafenib

All studies administered sorafenib orally at a standard dose of 400 mg twice daily. The timing of sorafenib initiation varied slightly across the studies but was generally started within a few days to a week after TACE. All studies mentioned that dose adjustments were made based on the patient’s tolerance and the presence of side effects. Sorafenib dosage could be reduced to 200 mg twice daily or temporarily discontinued if severe adverse events occurred. Regular monitoring of patients was conducted to assess liver function, blood counts, and adverse events. Imaging studies (CT or MRI) were performed to evaluate treatment response per Modified Response Evaluation Criteria in Solid Tumors (mRECIST) criteria [9–12].

Two TACE techniques are practiced: conventional TACE uses lipiodol and embolic agents, while the other employs drug-loaded microsphere beads with embolic properties. Both yield similar results in tumor response, progression time, OS, and safety [13]. Most studies included in this systematic review use the technique of lipiodol and embolic agents while Patidar et al. included both techniques.

Ren et al. are using 5-Fr catheters to identify tumour-feeding arteries for TACE techniques. Subsequently, Catheterization was aimed at segmental and subsegmental tumour-feeding arteries using a 2.3-Fr to 2.8-Fr tip microcatheter. The drug regimen was chosen by the doctor’s judgement either with oxaliplatin (50–100 mg) or pirarubicin (10–40 mg) with lipiodol (2–20 mL) chosen depending on tumor size. The drug was injected into the tumour-feeding arteries through the microcatheter. Gelatin sponge or polyvinyl alcohol particles were injected for embolization if necessary. Sorafenib started within 3 to 5 days after the initial TACE procedure [9].

In the Liu, CK study, TACE was performed using the modified Seldinger method and used angiographies to locate the blood vessels supplying the tumor. A micro-catheter was inserted into the blood vessels supplying the tumor; oxaliplatin was given at doses ranging from 100 to 200 mg, and acid glycosides were provided in amounts ranging from 500 to 1000 mg. Both *oxaliplatin* and glycosides can be given either separately or in combination. Following this, they continued administering epirubicin (30–60 mg) with 5–25 mL of iodized oil while observing the procedure with fluoroscopic monitoring. The administration of sorafenib began between the 4th and 7th day following the TACE procedure [10].

In Liu S’s study, the TACE procedure began with the femoral artery puncture, followed by the insertion of a catheter into the hepatic artery for targeted angiography. An identified artery supplying the tumor was injected with a mixture of lobaplatin, 5-fluorouracil, and poppy ethyl iodide oil. Sorafenib treatment was initiated between the 3rd to 5th days following each TACE procedure and temporarily stopped the day before each TACE session [11].

In the Patidar study, the TACE procedure involved using the Seldinger technique to access the common femoral artery. Arterial structure and tumor vascularization were assessed by angiography. A microcatheter was used for cannulation, followed by injection, and embolized with either a combination of epirubicin and lipiodol or epirubicin-loaded drug-eluting particles [12].

### Adverse event

In Ren et al.’s study, five individuals (8.2%) encountered delays in their treatment because of adverse effects resulting from TACE. Nonetheless, these patients initiated sorafenib therapy within a window of 6 to 14 days after concluding their TACE procedure. Additionally, 19 patients (31.1%) during treatment necessitated reductions in the prescribed sorafenib dosage, while five patients (8.2%) experienced interruptions in their medication regimen due to severe sorafenib-related adverse reactions. These adaptations were implemented to effectively manage the side effects and ensure the safety of the patients. The grading system used the National Cancer Institute Common Toxicity Criteria Adverse Events regarding severe adverse events. Fatigue was the most prevalent adverse event in the study receiving TACE-alone (19.0%). A minor percentage difference from fatigue, liver dysfunction is the second most frequent AE (18%). Conversely, in the group receiving both TACE and sorafenib, a wide range of adverse events was observed, which included hand-foot skin reactions (HFSR) as the most experienced by 75.4% of patients. Following diarrhea (47.5%) and liver dysfunction (32.8%), which ranked as the second and third most experienced. Regarding severe adverse events, they reported events that reached grade ¾ were HFSR by 18.0% of patients receiving TACE-sorafenib, followed by severe liver dysfunction in 13.1% of cases and severe diarrhea in 9.8%. Notably, no fatalities related to treatment were recorded in either of the study groups. The study findings indicated that incorporating sorafenib into the TACE therapy regimen was generally well-tolerated. Nonetheless, it is noteworthy that a significant portion of the patient population required adjustments in sorafenib dosage or experienced temporary treatment discontinuations due to adverse events [9]. Percentage of the adverse events described in Table 3.

**Table 3:** Percentage of adverse events

No.	Study (year)	Adverse event	TACE alone (%)	TACE-sorafenib (%)
1	Liu S et al. (2022)	Not mention	Not mention	Not mention
2	Ren et al. (2019)	Gastrointestinal bleeding	2	3.3
		Alopecia	0	31.1
		Hypertension	0.8	16.4
		HFSR	0	75.4
		Diarrhea	1.2	47.5
		Fatigue	19.0	24.6
		Liver dysfunction	18.2	32.8
3	Patidar et al. (2021)	Post-embolization syndrome	55	60
		Transient hepatic dysfunction	11	11
		HFSR	0	54.0
		diarrhoea	0	29.7
4	Liu CK et al. (2020)	Oral mucositis	5.0	71.4
		Hand-foot skin reaction	2.5	77.1
		Hypertension	5.0	34.3
		Alopecia	25	37.1
		Fatigue	57.5	68.6
		Liver function lesion	22.5	17.1
		Fever	70	69
		Leukopenia	40	57
		Diarrhea	52.5	48.6
		Nausea	55	60

Patidar et al. post-embolization syndrome, characterized by symptoms like fever, upper abdominal discomfort, nausea, and decreased appetite, was the most common consequence after TACE treatment. It mostly happened in the TACE-sorafenib group of patients (60%) and also in the TACE-only group, affecting (55%) of patients. Both study groups experienced transient hepatic dysfunction, as revealed by anomalies in liver function and a rise in ascites or emerging new ascites, impacting approximately 11% of the patients. Most importantly, this hepatic dysfunction gradually improved during the hospital stay following the procedure, returning to baseline levels within a week. As for sorafenib usage, the primary side effect was HFSR, seen in 54% of patients, followed by diarrhea, reported by 29.7%. Any required adjustments to sorafenib dosages were appropriately managed on an outpatient basis. Notably, neither study group encountered fatal adverse effects beyond these [12].

Liu, CK et al. conducted a comparison of both groups regarding adverse events reported in this study. Significant differences in adverse events became apparent. First, oral mucositis, characterized by mouth sores, was considerably more frequent in the TACE-sorafenib group, impacting 71.4% of patients, compared to a mere 5% in the TACE-alone group. This dissimilarity held high statistical significance (*P* < 0.001). Likewise, HFSR was notably more prevalent in the TACE-sorafenib group, affecting 77.1% of patients, while in the TACE-alone group, only 2.5% experienced it (*P* < 0.001). Moreover, hypertension was significantly more common in the TACE-sorafenib group, affecting 34.3% of patients, whereas in the TACE-alone group, only 5% of patients developed hypertension (*P* = 0.001) [10].

Regarding sorafenib-related adverse events, Liu CK et al. noted that eight patients had lowered the dosage of sorafenib to 200 mg twice daily because of an inability to tolerate it. Fortunately, there were no reports of severe adverse events. Significant relief of symptoms was achieved through symptomatic treatment, which aligns with what Ren et al. reported. It was also noted that 19 patients (31.1%) needed the dose to be reduced, and 5 patients (8.2%) had to temporarily stop taking the drug due to adverse events related to sorafenib [9, 10].

### Overall survival

The Patidar report showed significantly higher OS. Whereas, the TACE-sorafenib (10.1 months) and TACE-alone (7.8 months); thus, the superiority of combination therapy in terms of OS was pointed out with a *P* value < 0.001). The study of Ren et al. also reported OS TACE-sorafenib group median OS was 15.8 ± 2.0 months (95% CI 11.820–19.780); meanwhile, the TACE-alone group median OS was 7.8 ± 1.1 months (95% CI 5.607–9.993). Ren et al. showed a substantial difference in median OS between the TACE-sorafenib and TACE-alone groups, both in the BCLC-B and BCLC-C subgroups, with *p* values of 0.027 (HR = 0.547, 95% CI = 0.317–0.943) and 0.003 (HR = 0.507, 95% CI 0.320–0.801). However, these differences remained even after propensity score matching (PSM) with *P* values of BCLC-B 0.041(HR = 0.620, 95% CI 0.345–1.114) and BCLC-C 0.016 (HR = 0.544, 95% CI = 0.328–0.902), which reinforced the significant survival advantage the combination therapy [9, 12].

Liu CK et al. reported findings of overall survival of TACE-sorafenib therapy (13.6 months), with significantly better survival outcomes compared to the TACE-alone group (6.5 months), as represented by a *p* value of less than 0.05 for OS time (*P* = 0.041). The study by Liu S et al. emphasized the benefit of combining sorafenib with c-TACE, with a significant difference in OS, 22.9 months in the TACE-sorafenib group and 12.1 months in the TACE-alone group (*χ*2 = 5.848, *p* = 0.016) [10, 11].

Statistically, all studies were using SPSS software. The analysis of OS involved is Kaplan-Meier (KM), and the application of a log-rank test. Surprisingly, only one study presented the HR and CI of the Kaplan-Meier *p* values. none of the Kaplan-Meier data provided the number at risk or provided supplementary data so this systematic review could not perform a meta-analysis [9–12].

### Other parameters

Liu S et al. not only identified risk factors but also analyzed how factors affected patient survival in the two distinct treatment cohorts. In the TACE-sorafenib group, patients with portal vein invasion (*p* = 0.017), multiple TACE sessions (*p* = 0.021), liver cirrhosis (*p* = 0.040), and ascites (*p* = 0.013) experienced notably reduced survival rates. Similarly, in the TACE-alone group, having more than three tumors (*p* = 0.015), undergoing additional TACE sessions (*P* = 0.018), and ascites (*P* = 0.023) were recognized as risk factors associated with decreased survival. These findings underscore the importance of considering these factors when evaluating patient and treatment strategies.

Ren et al. also examined the BCLC-B subgroup, but this systematic review focused on BCLC-C. However, the result was also in line with the BCLC-C subgroup. Before PSM matching, the TACE-sorafenib group in BCLC-B had a median OS of 33.0 months with a confidence interval (CI) of 18.688–43.312 months, while the TACE-alone group median OS of 21.2 months with a CI of 16.696–25.704 months. After using propensity score matching (PSM), the median OS for the TACE-sorafenib group remained at 33.0 months with a CI of 18.688–43.312 months, while for the TACE-alone group, the median OS was 25.3 months with a CI of 13.135–39.465 months. Ren et al. performed multivariate analyses on the PSM cohort and discovered that treatment methods (*P* = 0.003), the number of nodules (*P* = 0.010), tumor size (*P* = 0.012), vascular invasion (*P* = 0.005), and the number of TACE procedures (*P* = 0.029) were all significant independent predictors of OS [9].

Moreover, Patidar et al. showed a significantly higher disease control rate (DCR) among the TACE-sorafenib therapy group (44.9%) compared to the TACE-alone group (25.8%) with a *p* value of 0.09, indicating better disease control in TACE-sorafenib group. Furthermore, the time-to-progression (TTP) of the TACE-sorafenib group was 4.6 months, in contrast to 3.1 months for the TACE-alone group (*P* = 0.001) [12].

Liu, CK et al. report TTP findings in the TACE-sorafenib group at 7.6 months which is better in comparison to the TACE-alone group at 3.4 months, with significantly better outcomes, *p* values of less than 0.05 for TTP (*P* = 0.002). One month into the treatment, tumor response was assessed using the mRECIST criteria and imaging follow-up. The combined treatment group also demonstrated a significant DCR compared to TACE-alone (*P* = 0.018), indicating that sorafenib enhances the effectiveness of TACE. Liu, S et al. also longer the progression-free survival (PFS) of combining sorafenib at 7.37 months with significant differences from TACE-alone at 5.97 months (*p* = 0.022). These combined outcomes underline the statistical significance of improved disease control and patient survival associated with the combination of sorafenib into TACE therapy across these studies [10, 11]. This indicates the superiority of combination therapy in terms of OS along with DCR, PFS, and TTP [9–12].

## Discussion

A definitive diagnosis of HCC is an absolute requirement. Patients must be confirmed to have HCC through histological examination, cytological tests, or imaging methods by established diagnostic criteria in all studies included in this review.

The current HCC BCLC-C recommendation is suitable for systemic palliative therapy as the only approach such as sorafenib. Even in the case of patients with inoperable HCC, sorafenib received approval based on the phase III, double-blind SHARP and Asia Pacific trials, both multicenter and placebo-controlled studies. Based on these trials [14]. A study by Abdel-Rahman et al. consisting of 41 patients with HCC with sorafenib therapy in Egypt showed 6.25 months as overall survival [15].

In general, TACE is typically treatment for patients meeting the criteria of BCLC-B stage, having an ECOG performance status of 2 or lower, and possessing Child-Pugh scores of either A or B. Even for BCLC-C patients with liver-dominant lesions and macrovascular invasion meeting the criteria of Child-Pugh A-B and a PS score of no more than 2, TACE remains the preferred recommended treatment [16]. All studies in this systematic review thoughtfully considered the liver function of the study population. Therefore, all study inclusion criteria consist of Child-Pugh class A or B and a PS score of no exceeding 2. Both studies by Ren et al. and Patidar et al. consider the Child-Pugh class and PS score as well as a serum bilirubin level below 3 mg/dl, and aspartate aminotransferase (AST) or alanine aminotransferase (ALT) levels within five times of normal [9, 12]. In A study by Zeeneldin et al. also conducted in Egypt, HCC patients with TACE therapy showed 16 months (95% CI 13–19 months) as overall survival [17].

In a comprehensive study conducted in the USA, Pawlik et al. assessed the safety and efficacy of a combined approach involving transarterial chemoembolization (TACE) and sorafenib in patients with advanced hepatocellular carcinoma (HCC). The findings revealed that the combination of sorafenib and DEB-TACE in individuals with unresectable HCC is well-tolerated and safe also demonstrated that most observed toxicities were associated with sorafenib, highlighting the manageability of such toxicity through dose adjustments of sorafenib [18]. In a UK-based study, Meyer et al. revealed that while the combination group displayed a noteworthy increase in overall survival, this disparity failed to attain statistical significance. The median overall survival stood at 631.0 days (95% CI 437.0–879.0) for the sorafenib group, compared to 598.0 days (500.0–697.0) for the placebo group (HR 0.91 [95% CI 0.67–1.24], *p* = 0.57). Given these findings, it is imperative to emphasize the need for further research to delve deeper into the factors influencing survival outcomes [19]. This systematic review is the first that uses a BCLC staging system comparing the OS of TACE-sorafenib to TACE-alone in BCLC-C. This systematic review follows the evolution of science, although the management of HCC stage C is limited.

Liu S et al. identified risk factors and analyzed how these factors affected patient survival in the two distinct treatment cohorts. The number of tumors of more than three is a risk factor affecting OS of TACE-Alone consistent with the Mishra et al. study. This factor also affects the OS of the TACE-sorafenib group consistent with Zheng et al. and Li et al. [20, 21]. The other finding such as ascites and liver cirrhosis as risk factors affecting overall TACE-sorafenib group therapy was not reported anywhere else. Portal vein invasion was found significant while Zheng et al. and Li et al. reported not significant [20, 21]. This finding stated the importance of considering these factors when evaluating patient and treatment strategies [11].

Most studies in this systematic review included patients with a history of TACE sessions [9–12]. Repeated TACE procedures should be cautiously approached due to their potential to cause liver damage and increased side effects. Consequently, the advantages and disadvantages must be thoroughly evaluated when contemplating additional TACE treatments. The fourth repeated TACE treatment did not exhibit a significant difference in survival with a *P* value = 0.21 [22]. In 2021, to make sure patients benefit from repeated TACE while potentially avoiding ineffective treatment., the JSH-Liver Cancer Study Group of Japan (JSH-LCSGJ) stated that the occurrence of intrahepatic invasion after one or two TACE sessions should be considered treatment failure. Based on this concept, the recommended course of action is to shift to a systemic agent in such cases [23]. However, Lu et al. stated that the combination of TACE and systemic therapy offers hope for patients with unresectable HCC, particularly those at risk of tumor recurrence after treatment or in cases of TACE failure or refractoriness [16]. In this systematic review, the advantages of TACE-sorafenib support this promising approach, which may encourage further research and guidelines in this field.

Three included studies reported that the most common sorafenib-related adverse event in the TACE sorafenib group was HFSR, followed by diarrhea and liver dysfunction. During treatment, some patients required reductions in the prescribed dose of sorafenib and experienced interruptions in their drug regimen due to severe sorafenib-related side effects. These adjustments were made to effectively manage side effects and ensure patient safety [9, 10, 12]. Meanwhile, one study did not mention any AEs [11]. One study also compared AEs in the TACE-sorafenib group to those in the TACE-alone group, and the results showed several AEs, including nausea, diarrhea, leukopenia, fever, liver function abnormalities, oral mucositis, fatigue, alopecia, and hand-foot skin reaction. However, only HFSR, hypertension, and oral mucositis showed a significantly higher prevalence in the TACE-sorafenib group compared to the TACE-alone group [10]. These findings emphasized the importance of considering these adverse events when deciding on treatment strategies. In line with Quinto et al. stated that complications are infrequent, and the incidence of mortality is low. However, they found major complications following the chemoembolization procedure were instances of decompensation leading to edema or ascites, acute cholecystitis, acute pancreatitis, liver rupture, liver abscess, and renal failure. Furthermore, post-embolization syndrome was noted in approximately 20% of the patients [24]. This systematic review finding is also in line with what Zhou et al. stated, there were no significant increases in liver-related adverse events or liver failure rates in the TACE-sorafenib group (*P* > 0.05). Li et al. also stated no fatalities in AEs occurred [21].

This systematic review reveals that the combination of TACE-sorafenib is superior in terms of OS. Likewise, Zou et al. stated the TACE-sorafenib group exhibited improved OS, with a mean of 32 months, compared to 21 months in the TACE-alone group (*P* = 0.0157) [25]. Conversely, Li et al. study did not improve OS significantly, the median OS was 48.5 months with TACE-sorafenib and 41.0 months with TACE-alone [21]. These offer valuable insight for healthcare practitioners seeking to enhance patient outcomes.

This systematic review also mentions another parameter that is superior in combination TACE-sorafenib group such as TTP, PFS, and DCR. This result is in line with Kudo et al. who showed a superior median PFS of 25.2 months compared to 13.5 months in the TACE-alone group (*p* = 0.006) [26] and a study conducted by Zou et al. stated PFS and DCR superiority of the TACE-sorafenib group, a mean PFS of 21 months, in contrast to 12 months (*P* = 0.0005). While DCR is statistically significant at 80.95% compared to 55.81% (*P* < 0.05) [25]. However, this finding is not the focus of this systematic review.

This study focuses exclusively on BCLC-C, and there are relatively few studies that address this specific comparative issue. During the process of this study, we found three studies lacked the number at risk and supplementary data, thus we couldn't perform a meta-analysis. Across the four studies included, there is a lack of diversity within the study population. here our included study is from the same race and ethnicity. To validate the findings of this study, future research should aim to include a more diverse range of patients representing multiple racial and ethnic backgrounds. Despite the limited research capabilities, the results of this systematic review are noteworthy because they show the potential of combination therapy as a superior management of treatment. Researchers must remain critical in finding superior therapies for HCC BCLC-C.

## Conclusion

In conclusion, this systematic review reported that TACE-sorafenib therapy improves overall survival (OS) in patients with HCC at BCLC stage C without significantly increasing adverse events compared with TACE-alone.

## Supplementary Information

**Supplementary Material 1.**

**Supplementary Material 2.**

## Data Availability

All data provided in the statement for this study are reported in a published article

## Abbreviations

AEs: Adverse events
BCLC: Barcelona Clinic Liver Cancer
CI: Confidence intervals
DCR: Disease control rate
ECOG: Eastern Cooperative Oncology Group
HCC: Hepatocellular carcinoma
HFSR: Hand-foot skin reaction
HR: Hazard ratio
mRECIST: Modified RECIST
NOS: Newcastle-Ottawa Scale
OS: Overall survival
PFS: Progression-free survival
PD: Progressive disease
PR: Partial response
PRISMA: Preferred Reporting Items for Systematic Review and Meta-analysis
RR: Risk ratio
TACE: Transarterial chemoembolization
TTP: Time to progression
VEGF: Vascular endothelial growth factor
